# Empagliflozin-activated AMPK elicits neuroprotective properties in reserpine-induced depression via regulating dynamics of hippocampal autophagy/inflammation and PKCζ-mediated neurogenesis

**DOI:** 10.1007/s00213-024-06663-0

**Published:** 2024-08-19

**Authors:** Radwa N. Muhammad, Mohammed A. Albahairy, Mai A. Abd El Fattah, Weam W. Ibrahim

**Affiliations:** Department of Pharmacology and Toxicology, Faculty of Pharmacy, Cairo University, Cairo, 11562 Egypt

**Keywords:** AMPK, Autophagy, Depression, Empagliflozin, Neurogenesis, PKCζ

## Abstract

**Rationale:**

Major depression has been an area of extensive research during the last decades, for it represents a leading cause of disability and suicide. The stark rise of depression rates influenced by life stressors, economic threats, pandemic era, and resistance to classical treatments, has made the disorder rather challenging. Adult hippocampal neurogenesis and plasticity are particularly sensitive to the dynamic interplay between autophagy and inflammation. In fact, the intricate balance between the two processes contributes to neuronal homeostasis and survival.

**Objectives:**

Having demonstrated promising potentials in AMPK activation, a major metabolic sensor and autophagy regulator, empagliflozin (Empa) was investigated for possible antidepressant properties in the reserpine rat model of depression.

**Results:**

While the reserpine protocol elicited behavioral, biochemical, and histopathological changes relevant to depression, Empa outstandingly hindered these pathological perturbations. Importantly, hippocampal autophagic response markedly declined with reserpine which disrupted the AMPK/mTOR/Beclin1/LC3B machinery and, conversely, neuro-inflammation prevailed under the influence of the NLRP3 inflammasome together with oxidative/nitrative stress. Consequently, AMPK-mediated neurotrophins secretion obviously deteriorated through PKCζ/NF-κB/BDNF/CREB signal restriction. Empa restored hippocampal monoamines and autophagy/inflammation balance, driven by AMPK activation. By promoting the atypical PKCζ phosphorylation (Thr403) which subsequently phosphorylates NF-κB at Ser311, AMPK successfully reinforced BDNF/CREB signal and hippocampal neuroplasticity. The latter finding was supported by hippocampal CA3 toluidine blue staining to reveal intact neurons.

**Conclusion:**

The current study highlights an interesting role for Empa as a regulator of autophagic and inflammatory responses in the pathology of depression. The study also pinpoints an unusual contribution for NF-κB in neurotrophins secretion *via* AMPK/PKCζ/NF-κB/BDNF/CREB signal transduction. Accordingly, Empa can have special benefits in diabetic patients with depressive symptoms.

**Limitations:**

The influence of *p*-NF-κB (Ser311) on NLRP3 inflammasome assembly and activation has not been investigated, which can represent an interesting point for further research.

**Graphical abstract:**

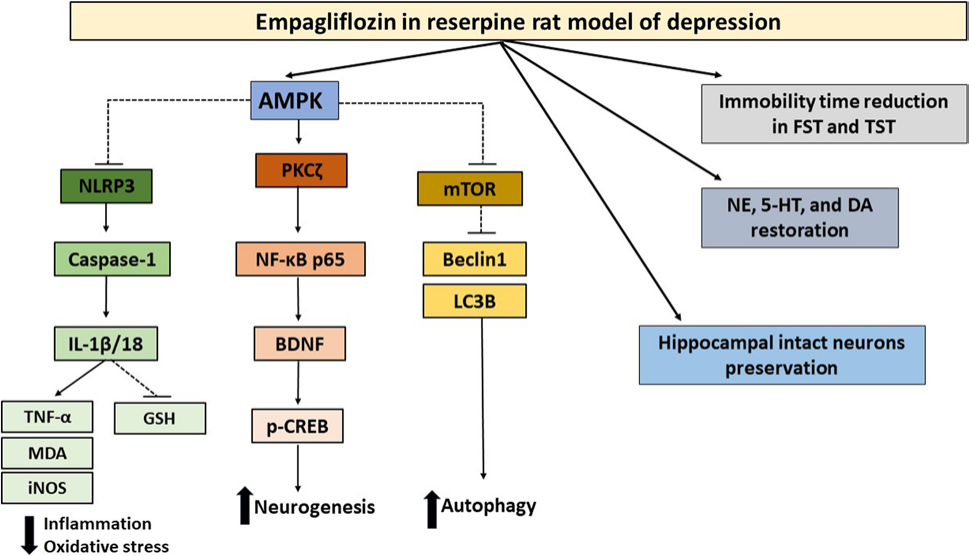

**Supplementary Information:**

The online version contains supplementary material available at 10.1007/s00213-024-06663-0.

## Introduction

Depression is one of the most common neuropsychiatric disorders and a principal reason of disability worldwide. According to the World Health Organization estimation, depression affects globally more than 264 million people of all ages (Hanifiha et al. 2022; James et al. 2018). The disorder is highly heterogeneous with clinical manifestations characterized by a triad of emotional, neurovegetative, and neurocognitive symptoms which considerably deteriorate patients’ quality of life and social functioning (Park et al. 2018). Suicidal ideation and attempts are further attributes of depression, where up to 15% of severely depressed patients commit suicide. Additionally, depression has long been linked with developing other cardiovascular, metabolic, and central nervous system (CNS) diseases (Cizza et al. 2001; Hoerster et al. 2019).

The most accepted theories of depression include the historic monoamine neurotransmission deficiency, impaired hippocampal neurogenesis, and abnormal synaptic plasticity hypotheses, in addition to a subsidiary role for oxidative stress and neuro-inflammation (Jeon and Kim 2016; Liu et al. 2020). Nonetheless, significant concerns have been raised against the monoamine theory which left behind many unanswered questions. Importantly, this theory does not explain why many patients are considered "partial responders" to traditional antidepressants, and some are rendered with "treatment-resistant depression" (Otte et al. 2016; Pitsillou et al. 2020). Accordingly, it sounds imperative to search for new therapies that delve beyond monoamines and that address the underlying pathophysiology of depression. Given the well-established role of the monoamine theory, different animal models have been replicated to produce a depressive phenotype through depletion of central monoamines. Reserpine (Res), a no longer used antihypertensive alkaloid drug, acts mainly by depleting catecholamine stores in the CNS by acting as an irreversible inhibitor of the vesicular amine pump (Ikram and Haleem 2019). Therefore, single and repeated administration of Res is utilized as an animal model that mimics depression (Antkiewicz-Michaluk et al. 2014).

Although neuro-inflammation has been strongly associated with neurodegenerative disorders (Borikar et al. 2022), its significant contribution in depression and other neuropsychiatric diseases is still under debate (Hassamal 2023; Hurley and Tizabi 2013). In fact, the inflammatory response can start in the periphery and culminate in the brain or break out within the CNS under certain stimuli. Despite being a tightly regulated process that can be rather adaptive, negative aspects of neuro-inflammation lead to neurodegeneration and alterations in synaptic plasticity, resulting in dysfunction of emotion processing (Cai et al. 2019; Llorens-Martín et al. 2014). The nod-like receptor protein 3 (NLRP3) inflammasome is the most protein complex expressed during inflammation; motivating the secretion of interleukin (IL)-18 and IL-1β *via* caspase-1 activation (Cao et al. 2019; Tian et al. 2021). These released ILs have been strongly associated with stress response and depression by means of inhibiting hippocampal neurogenesis and disrupting neural networks that are involved in reward processing (Hassamal 2023). Accordingly, it is postulated that evidence of neuro-inflammation in depressed patients reflects poor prognosis and response to treatment (Troubat et al. 2020). It has been also believed that antidepressants exert their clinical benefit partly through hindering the NLRP3 inflammasome cascade by stimulating cellular autophagy (Trojan et al. 2019). Autophagy is a vital process for cellular homeostasis and it has been linked to several pathologies, including those which are stress-related (Gassen and Rein 2019). Just like inflammation, autophagy is a strictly controlled endeavor which is mainly responsive to nutrient and growth factors-sensitive pathways, like adenosine monophosphate-activated protein kinase (AMPK) and mechanistic target of rapamycin (mTOR), the master regulator of autophagy. AMPK is a pivotal adjuster of autophagy-related proteins, including microtubule-associated protein light chain 3 (LC3B) and Beclin1. In the same context, both pharmacological and non-pharmacological interventions have been shown to trigger different autophagic pathways to modulate depression-associated pathological mechanisms and behavior (Tang et al. 2021). It is worth noting that the autophagy protein LC3B has been negatively correlated with NLRP3 inflammasome activation in the spinal cord of mice with autoimmune encephalomyelitis (Shao et al. 2014), and in cerebral ischemia/reperfusion injury (Wang et al. 2019). Furthermore, reactive oxygen species (ROS) are known to thrive when autophagy deteriorates (Trocoli and Djavaheri-Mergny 2011). Interestingly, activation of AMPK has been reported to ameliorate depressive-like behavior *via* activating key neurogenesis pathways (Odaira et al. 2019). Nonetheless, besides to the chief role of AMPK and mTOR in orchestrating autophagy, complex interplay between nuclear factor kappa B (NF-κB) and autophagic assembly has been suggested (Youssef et al. 2021). In fact, due to its well-established role in mediating inflammation, it sounds relevant that NF-κB can repress autophagy (Singh and Singh 2020). Nevertheless, studies have revealed that the two players can control each other through positive and negative feedback mechanisms to achieve homeostasis. In addition, it has been concluded that NF-κB can indirectly activate or inhibit autophagy depending on the driving stimulus and cellular context (Mattson and Camandola 2001; Zhang and Hu 2012). Moreover, in the CNS, NF-κB plays a critical role in the coordinate expression of anti-apoptotic and anti-inflammatory genes, which promotes neuronal survival and, on the contrary, its inhibition causes neuronal death and neurodegenerative milieu (Koulich et al. 2001). In the same context, NF-κB is a potent inducer of Beclin1 expression; where suppression of NF-κB could disturb the expression of Beclin1, resulting in impairment of phagocytic clearance of neuritic plaque (Copetti et al. 2009). Most importantly, if phosphorylated at Ser311, NF-κB mediates the secretion of two critical neurotrophic factors: the nerve growth factor and brain-derived neurotrophic factor (BDNF) under the influence of protein kinase C (PKC)—an outcome that enhances neurogenesis and neuroplasticity (Ji et al. 2010; Lin et al. 2014; Obara et al. 2001).

Empagliflozin (Empa) is a novel oral anti-hyperglycemic agent that selectively inhibits sodium − glucose cotransporter-2 (SGLT2) in proximal renal tubules, thus promoting urinary glucose excretion and overcoming hyperglycemia, which is independent from insulin (Steven et al. 2017). Acknowledging the fact that SGLT2 inhibitors (gliflozins) can access the brain where their molecular target has been identified, research attention has been directed towards the extra cardiovascular and renal benefits of these drugs. However, cumulative evidence has revealed that SGLT2 inhibitors can modify various cellular, metabolic, and bioenergetic mechanisms to amend disease-associated pathological changes in the CNS (Pawlos et al. 2021; Rizzo et al. 2022). Apart from SGLT2 inhibition, gliflozins have been reported to activate AMPK and to replenish autophagy in various organs to achieve cellular protection and homeostasis (Chen et al. 2023; Fukushima et al. 2021; Packer 2022; Safaie et al. 2024). In recent years, growing studies have been unmasking significant neuroprotective properties of SGLT2 inhibitors in different CNS-related disorders, such as Alzheimer’s disease, Huntington's disease, Parkinson's disease, as well as cerebral ischemia, major depression, and epilepsy (Borikar et al. 2024; El-Sahar et al. 2020; Muhammad et al. 2021; Rizzo et al. 2022). In addition, Empa outstandingly refined cognition in a murine mixed model of diabetes and Alzheimer’s disease. The latter effect was linked to its ability to reduce neuronal loss, brain atrophy and hemorrhage, as well as microglial burden and amyloid plaques (Hierro-Bujalance et al. 2020). Moreover, Empa could mitigate cerebral ischemia/reperfusion-induced neuronal degeneration in hyperglycemic rats with consequent neuroprotective effects, which might be attributed not only to glycemic control, but also to its antioxidant, anti-inflammatory, and antiapoptotic properties (Amin et al. 2020). Interestingly enough, in a very recent case–control observational study, the authors have yielded unexpected outcomes, where the use of gliflozins in diabetic patients was associated with higher risks of depression and cognitive decline when compared to the control group (Nodirahon et al. 2024). However, such conflicting studies are rather limited and further investigations are mandatory to support or deny these assumptions.

The current study aims at investigating the effect of Empa in a Res model of depression in rats. It focuses on evaluating the dynamic interplay between autophagy and neuro-inflammation in the pathogenesis of depression. Moreover, the study unmasks the promising role of Empa as a fine-tuning agent that can regulate both processes to achieve neural resilience.

## Materials and methods

### Animals

This research was implemented according to the principles of The Guide for Care and Use of Laboratory Animals published in (2011) and was accepted by the Ethical Committee for Animal Experimentation of the Faculty of Pharmacy, Cairo University (Permit number: PT 3108). All efforts were exerted to minimize animals' suffering during the investigation duration.

Adult male Wistar rats, weighing 180 − 200 g, were obtained from the animal facility of the Egyptian Drug Authority (Giza, Egypt). Rats were adapted to the animal facility circumstances for 2 weeks before the beginning of the study. Animals were conserved under controlled environmental conditions of temperature (23 ± 2 °C), humidity (60 ± 10%), and light/dark (12/12 h) cycle. Food and water were allowed without hindrance during the experimental period.

### Drugs and chemicals

In the current investigation, Res, escitalopram (Esc), and Empa were obtained from Sigma-Aldrich Chemical Co. (MO, USA), Apex Pharmaceutical Co. (Cairo, Egypt), and Boehringer Ingelheim Pharmaceuticals (CT, USA), respectively. The drugs were freshly prepared daily by dissolving in saline just before administration. All other chemicals were of the highest analytical grade.

### Experimental design

**Fig. 1:**
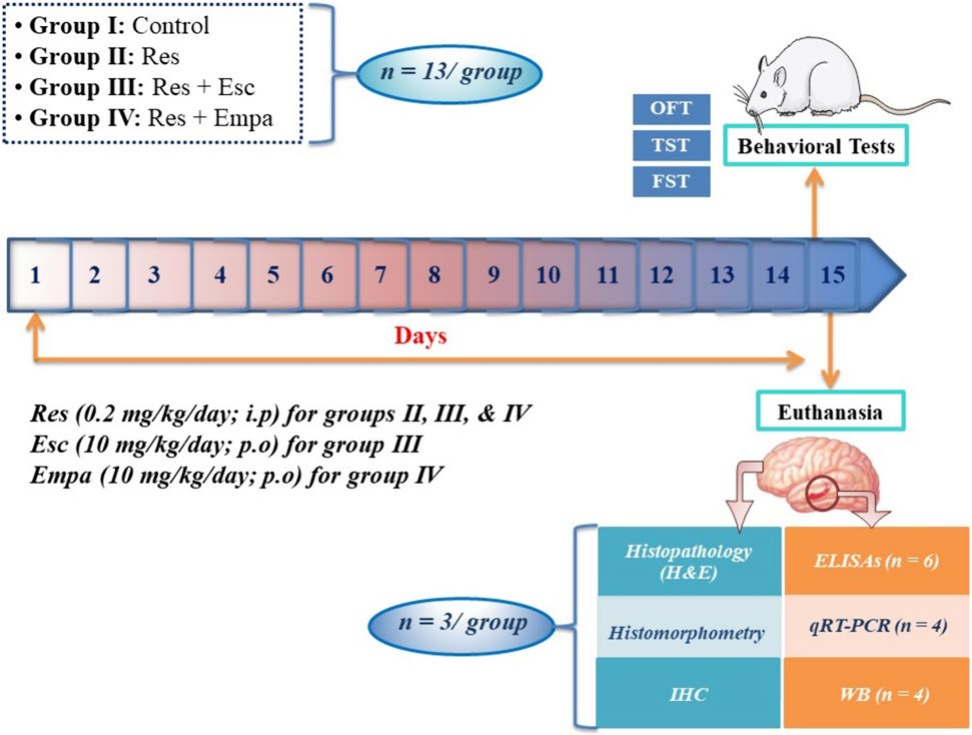
Graphical illustration for the experimental timeline. Empa; empagliflozin, Esc; escitalopram, FST; forced swimming test, H & E; hematoxylin & eosin, IHC; immunohistochemistry, OFT; open field test; qRT-PCR; quantitative (real-time) reverse transcription polymerase chain reaction, Res; reserpine, TST; tail suspension test, WB; western blotting

As shown in Fig. 1, rats were randomly assigned into 4 groups (n = 13/group) as follows: Group I received an appropriate volume of saline intraperitoneally (i.p) and served as the negative (control) group. In group II, Res was given i.p at a dose of 0.2 mg/kg/day for 14 successive days, which counted as the positive control (model) subset. The protocol of Res administration for inducing a depressive phenotype was chosen based on prior studies (Antkiewicz-Michaluk et al. 2014; Khadrawy et al. 2018). Rats in group III received Esc (10 mg/kg/day) orally (Waugh and Goa 2003); one hour before Res injection. Finally, in group IV, rats received oral doses of Empa (10 mg/kg/day); one hour before receiving Res. The Empa dose (10 mg/kg) was selected based on prior studies (Abdel-latif et al. 2020; Amin et al. 2020; Hierro-Bujalance et al. 2020). Empa has been reported to alleviate neuronal apoptosis and enhance neurobehavioral functions in a dose-dependent manner in cerebral/ischemia reperfusion-injured rats, where the higher dose (10 mg/kg) was more protective than the lower one (1 mg/kg) (Abdel-latif et al. 2020). Treatment with Empa (10 mg/kg; i.p) also showed significant amelioration of behavioral/neurological functions and histopathological changes observed in brain tissues of hyperglycemic rats subjected to cerebral ischemi/reperfusion injury *via* suppressing oxidative/inflammatory/apoptotic pathways (Amin et al. 2020). In addition, it has been reported that Empa (10 mg/kg) attenuated neuronal damage, hemorrhage, and microglial burden, as well as cognitive deficits in a mixed murine model of Alzheimer’s disease and type 2 diabetes (Hierro-Bujalance et al. 2020).

Drugs administration lasted for 14 days and, on the 15^th^ day, animals were subjected to behavioral assessments to verify the depressive phenotype. Eventually, rats were scarificed by decapitation under anesthesia using phenobarbitone (40 mg/ kg; i.p) (El-Sahar et al. 2020).

For histopathological investigations, whole brain tissues of 3 rats/group were directly immersed in 10% phosphate-buffered formalin solution until being processed. Meanwhile, the hippocampi of 10 rats/group were rapidly dissected and subdivided into two subsets; the first subset (n = 6/group) was used to prepare 10% hippocampal homogenates using phosphate-buffered saline, and the second subset (n = 4/group) was used for subsequent western blotting and PCR assessments. All hippocampal specimens were kept at -80° C and only thawed at the time of the respective analysis.

### Behavioral assessments

After 24 h of the last injection day, locomotor function of the animals was evaluated through the open field test (OFT), and they were examined for their depressive behavior using tail suspension and forced swimming tests. All tests were performed in a sound-isolated room starting at 12 p.m. with a two-hour resting period between the tests.

#### Open Field Test (OFT)

The OFT was accomplished to evaluate spontaneous locomotor function of rats using a square wooden box (80 × 80 × 40 cm) with red walls and a black polished floor divided by white lines into 16 equal squares (4 × 4). Individually, animals were placed gently into the center of the open field apparatus and the behavior of each rat was recorded for 3 min using an overhead camera existing in the room. The floor and walls were cleaned after each testing session using 70% ethyl alcohol to remove possible odors left by prior rats. The ambulation frequency, which is the number of times each rat crosses one of the grid lines with all four paws, was assessed (Khadrawy et al. 2018; Yu et al. 2019).

#### Tail Suspension Test (TST)

The TST was implemented to assess behavioral despair by measuring the immobility time displayed by rats. Each rat was fixed upside down about 50 cm above the floor using adhesive tape placed approximately 1 cm from the tip of the tail in such a position that rendered rats unable to escape or to hold on to nearby surfaces. Rats were considered immobile when they stopped struggling to overcome this abnormal position or when they became completely motionless after a period of struggling activity. This test was performed for 5 min and the total duration of immobility was recorded (Takahashi et al. 2018).

#### Forced Swimming Test (FST)

The FST is a widely used assessment tool for the effect of antidepressants in animal models of depression. In this test, rats were placed individually into a glass cylinder (height: 50 cm; diameter: 20 cm) containing tap water (25 ± 2 °C; depth: 30 cm). These criteria should not allow the animals to support themselves by touching the bottom of the cylinder with their paws or tails. The assessment lasted for 5 min and, after cessation of vigorous activity by rats, the total duration of immobility was recorded with the help of a stopwatch. Rats were considered immobile when they stopped swimming; making only the necessary movements for keeping their heads above the water surface. Of note, swimming water was regularly changed between sessions (Han et al. 2009; Takahashi et al. 2018).

### Biochemical measurements

#### Enzyme-Linked Immunosorbent Assays (ELISAs)

MyBiosource (CA, USA) specific ELISA kits were purchased for determination of hippocampal contents of serotonin (5-HT; Cat. No. MBS9362408), dopamine (DA; Cat. No. MBS7214676), reduced glutathione (GSH; Cat. No. MBS265966), malondialdehyde (MDH; Cat. No. MBS727531), inducible nitric oxide synthase (iNOS; Cat. No. MBS263618), caspase-1 (Cat. No. MBS765838), IL-1β (MBS825017), *p*-PKC zeta (ζ; Thr403; Cat. No. MBS725403), and BDNF (Cat. No. MBS824814), each according to the manufacturer's instructions.

Additionally, hippocampal contents of noradrenaline (NE) and tumor necrosis factor (TNF)-α were measured using Cusabio ELISA kits (Wuhan, China; Cat. No. CSB-E07022r and CSB E11987r, respectively), and that of IL-18 was estimated by the specific ELISA kit obtained from Elabscience (Wuhan, China; Cat. No. E-EL-R0567). Meanwhile, the protein content of each sample was detected in the aliquots according to the method of Lowry et al. (2014).

#### Western blot analysis

Estimation of the phosphorylated forms of AMPK (Thr172) and cAMP response element-binding protein (CREB; Ser133), as well as NF-κB p65 (Ser311), and finally mTOR hippocampal protein expressions were accomplished using western blot analysis. The right-sided hippocampi of 4 rats per group were homogenized using ReadyPrep™ protein extraction kit (Bio-Rad Inc., CA, USA). Following protein quantification using Bradford protein assay kit (ThermoFisher Scientific Inc., MA, USA), equal protein amounts from each sample were separated by sodium dodecyl sulfate–polyacrylamide gel electrophoresis (SDS-PAGE) and then transferred to a nitrocellulose membrane using a semi-dry transfer apparatus (Bio-Rad, CA, USA). The membranes were then blocked with 3% bovine serum albumin (BSA) in Tris-buffered saline containing Tween 20 (TBST) at room temperature for 1 h to prevent non-specific protein binding. Afterwards, the membranes were incubated overnight at 4 °C on a roller shaker with the corresponding primary antibodies: anti-β-actin (Cat. No. MA1-91,399), anti-*p*-AMPK (Thr172; Cat. No. PA5-37,821), anti-*p*-CREB (Ser133; Cat. No. MA5-11,192), anti-*p*-NF-kB p65 (S311; Cat. No. PA5-104,959), and anti-mTOR antibodies (Cat. No. AHO-1232). All the primary antibodies were obtained from ThermoFisher Scientific (MA, USA). Subsequently, the generated blots were rinsed 3 − 5 times for 5 min with TBST and incubated for 2 h at room temperature with horseradish peroxidase-conjugated secondary antibody (Dianova, Hamburg, Germany). Finally, the chemiluminescence substrate reaction was applied to the blot according to the manufacturer's recommendation (Amersham Biosciences, IL, USA). The corresponding intensities of the established protein bands were measured using densitometric analysis by the aid of a scanning laser densitometer (Biomed Instrument Inc., CA, USA). The results were represented as arbitrary units related to β-actin bands’ intensities.

#### Quantitative real-time PCR analysis of NLRP3

All samples were assayed in duplicates, where total RNA was extracted from left-sided hippocampal tissues (n = 4/group) using SV Total RNA Isolation System (ThermoFisher Scientific, MA, USA). RNA purity was estimated by 260/280 nm absorption ratio using ThermoFisher Scientific NanoDrop™ spectrophotometer. Using 1 μg of the extracted RNA per sample, reverse transcription (RT) of RNA into complementary DNA (cDNA) was developed by the aid of the Reverse Transcription System (Promega, Leiden, Netherlands) in accordance with the manufacturer's guidelines. NLRP3 gene expression was determined *via* quantitative (q) real-time PCR using Maxima SYBR Green qPCR Master Mix (ThermoFisher Scientific, MA, USA), where 5 μL cDNA was used and exposed to 40 cycles of denaturation at 95 °C for 15 s, annealing at 60 °C for 60 s, and extension at 72 °C for 60 s. The specific primers and probes were chosen from Applied Biosystems TaqMan Assays inventories. The sequences of the used primers are listed in Table 1. Data quality check was performed using amplification, primer melting temperature, and cycle threshold values to exclude any outliers before calculation. The 2^−ΔΔCT^ formula was used to obtain the expression of target genes relative to the housekeeping gene, β-actin (Livak and Schmittgen 2001).

**Table 1:** Primer sequences used for qRT-PCR

mRNA species	Primer sequence (5'‒3')
NLRP3	F: TGCCCGTCTG GGTGAGA
	R: CCGGTGCTCCTTGATGAGA
β-actin	F: GCTGTCTGCCTTGGTAGTGGAT
	R: GCATCGTCACCACCAAAGC

### Histopathological inspections

The brains of 3 rats per group were removed and fixed in 10% buffered formalin for 72 h. Afterwards, the specimens were washed, dehydrated in serial grades of ethanol, and cleared in xylene. Samples were then infiltrated and embedded into Paraplast tissue embedding media (Leica Biosystems, Wetzlar, Germany). Serial sagittal brain sections of 5 μm thickness were cut by a rotatory microtome and stained with hematoxylin and eosin (H & E) for histopathological examination of the hippocampal regions under light microscope. For detecting surviving neurons, 5 μm-thick paraffinized sections were stained with a toluidine blue stain, then seven random non-overlapping fields/tissue section were examined for quantification of intact neurons in the hippocampal Cornu Ammonis (CA3) region. All micrographs were obtained using a full HD microscopic imaging system operated by Leica application module for histological analysis (Leica Microsystems GmBH, Wetzlar, Germany). All standard procedures and protocols for sample fixation and staining were carried out according to the previously described method (Culling 2013). Histopathological handling and assessment of specimens were accomplished by a professional observer who was unaware of samples identity to avoid any bias.

### Immunohistochemical examination of Beclin1 and LC3B

Immunohistochemical staining for Beclin1 and LC3B was done as stated in the manufacturer’s guidelines. Four μm-thick paraffinized tissue sections were first dewaxed with xylene, then rehydrated in graded ethanol, and finally heated for 5 min in citrate buffer. Hydrogen peroxide (0.3%) was used to block antigen-retrieved brain tissue sections that were then incubated with the primary antibodies; anti-Beclin1 (1:100; GenTex, CA, USA; Cat. No. GTX55535) and anti-LC3B antibody (1:200; Bioss Antibodies, MA, USA; Cat. No. bs-11731R). Thereafter, sections were washed with phosphate-buffered saline and incubated for 20 min with the biotinylated link antibody and peroxidase-labelled streptavidin (Dako Carpinteria, CA, USA). The reaction was visualized with diaminobenzidine tetrahydrochloride (DAB) substrate kit (Vector Laboratories Inc., CA, USA). Counter staining with hematoxylin was accomplished, followed by dehydration and clearing in xylene. Tissue sections were then slipcovered for microscopic examination.

For immunohistochemical quantitative analysis, six random non-overlapping fields/immunostained tissue section were analyzed for determining Beclin1 and LC3B levels. All morphological examinations, photographs, as well as quantitative analysis were recorded using Leica's application system modules for histological analysis (Leica Microsystems GmbH, Wetzlar, Germany).

## Statistical analysis

Results are illustrated as means ± SD. One − way ANOVA followed by Tukey’s multiple comparison test were used for analyzing all results. Statistical analysis was accomplished using GraphPad Prism software (version 9) with the probability level of significance less than 0.05. For each effect, the F-value (F), degrees of freedom (df), the effect size partial eta squared (ηp^2^), and statistical significance (p) were reported. Additionally, pairwise comparisons were provided in each relevant figure.

## Results

### Effect of Empa on Res-induced alterations in OFT, TST, and FST

**Fig. 2:**
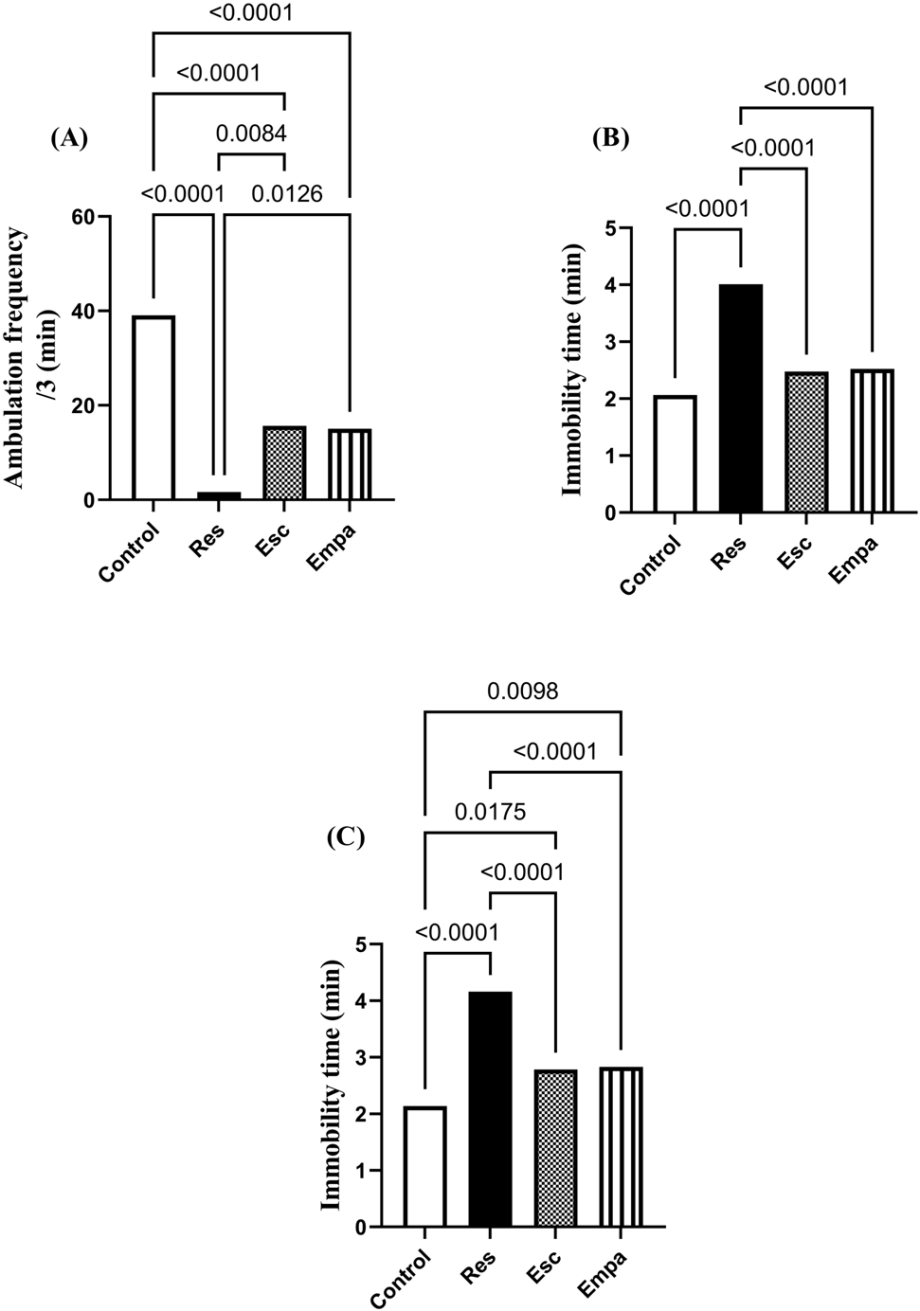
Effects of Empa on Res-induced alterations in (A) ambulation frequency in the OFT, (B) immobility time during the TST, and (C) immobility time during the FST. Data is expressed as mean ± SD (n = 13), using one-way ANOVA with Tukey − Kramer multiple comparison post-test, (*p* < 0.05). Empa; empagliflozin, Esc; escitalopram, FST; forced swimming test, OFT; open field test, Res; reserpine, TST; tail suspension test

Rats exposed to i.p Res injections exhibited behavioral alterations signifying depression-like symptoms, which was reflected in the considerable prolongation of immobility durations in TST as well as FST, when compared to the control group (for immobility duration in TST: F_(3, 48)_ = 18.85, ηp^2^ = 0.5409, p < 0.0001 and for immobility duration in FST: F_(3, 48)_ = 32.42, ηp^2^ = 0.6695, p < 0.0001). Treatment with either Esc or Empa showed equivalent efficacy in terms of decreasing the immobility periods and restoring them to the normal values (Fig. 2B & C).

Of note, rats subjected to Res injection showed lower ambulation frequency in the OFT as compared to the control group (F_(3, 48)_ = 27.59, ηp^2^ = 0.6330, p < 0.0001). Moreover, no significant difference was elucidated in the number of squares crossed during the 3-min test when treating rats with Esc or Empa, as related to the Res group (Fig. 2A).

### Effects of Empa and Res on hippocampal histopathology and histomorphometry

**Fig. 3:**
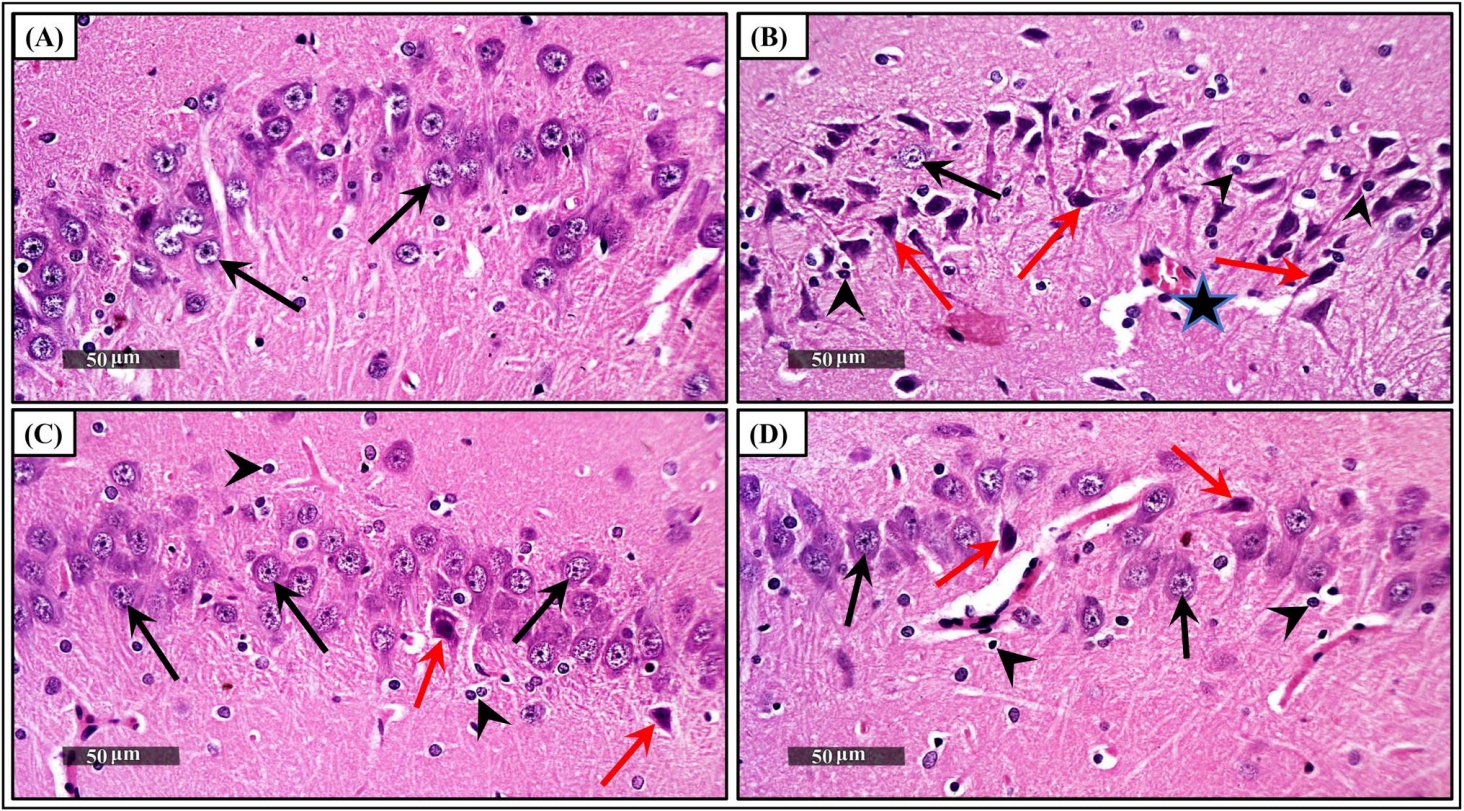
Effects of Empa and Res on hippocampal histopathology. Representative H & E-stained photomicrographs (CA3 region, n = 3, 400 ×) for: (A) panel from the control group demonstrating normal organized morphological structures of hippocampal layers *(black arrows),* intact intercellular matrix, and minimal reactive glial cell infiltrates. (B) The Res group reveals severe neuronal degeneration, abundant records of hyperesenophilic, pyknotic, and structureless pyramidal neurons *(red arrows),* few dispersed apparent intact cells *(black arrows),* severe perineuronal edema, high reactive glial cell infiltrates *(arrowheads),* along with congested blood vessels *(star).* Sections from (C) Esc and (D) Empa showing marked regression of the neuropathological alterations signified by minimal sporadic records of degenerated neurons *(red arrows),* relatively higher figures of intact neurons *(black arrows),* and milder persistent records of reactive glial cell infiltrates *(arrowheads)* along with normal appearance of brain matrix. Empa; empagliflozin, Esc; escitalopram, Res; reserpine

**Fig. 4:**
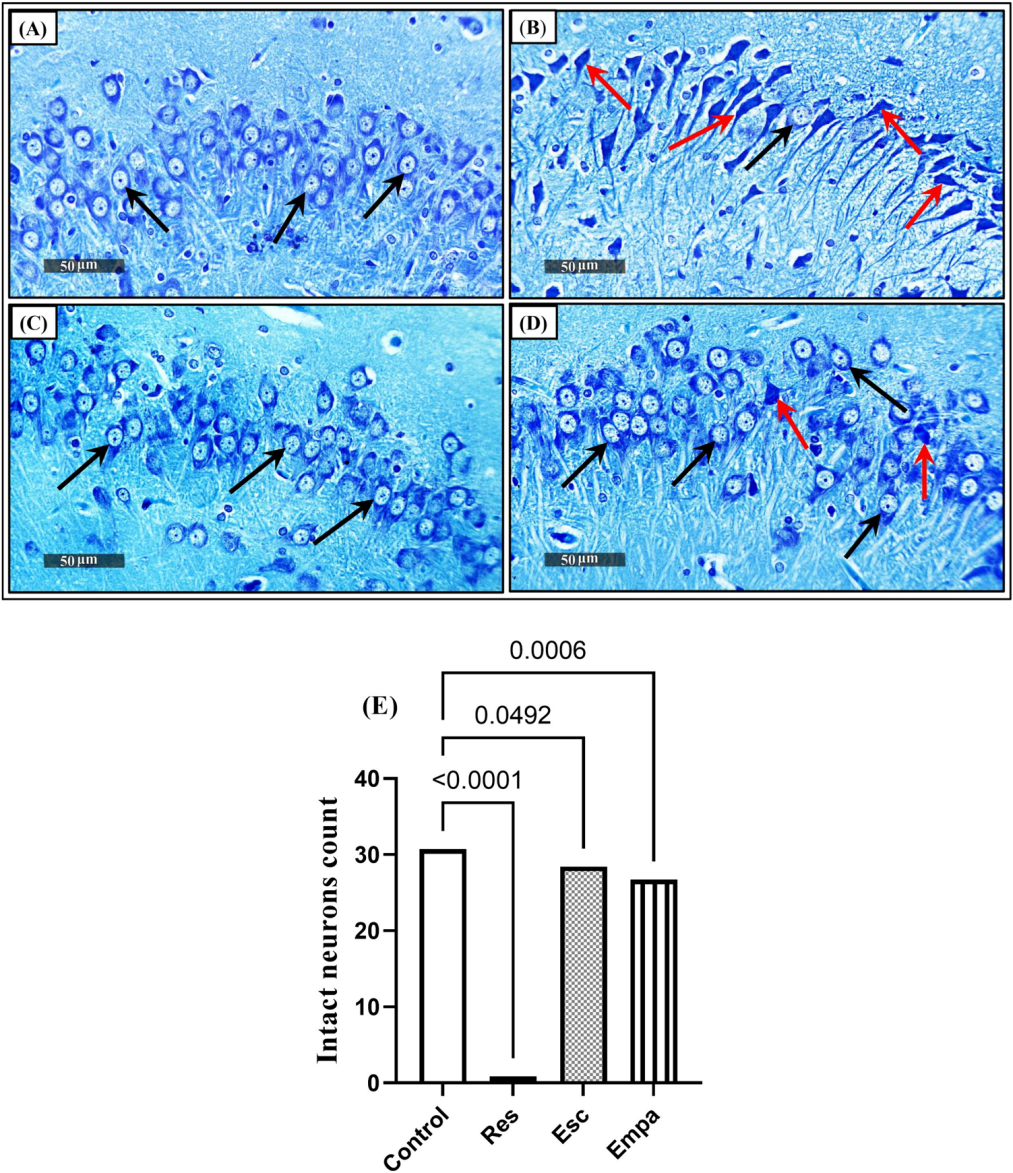
Effects of Empa and Res on hippocampal histomorphometry. Illustrative toluidine blue-stained photomicrographs (CA3 region, n = 7, 400 ×) from (A) control, (B) Res, (C) Res + Esc, and (D) Res + Empa-treated groups. Black arrows represent intact neurons, whereas red arrows indicate degenerated ones. The number of intact neurons per group is represented in panel (E), where data is presented as mean ± SD using one-way ANOVA followed by Tukey's post-hoc test, (*p* < 0.05). Empa; empagliflozin, Esc; escitalopram, Res; reserpine

Microscopic examination of H & E brain sections (Fig. 3) was carried out in addition to using toluidine-blue stain (Fig. 4) to determine the mean count of intact neurons in CA3 hippocampal areas. While the control group demonstrated normal organized morphological structures of hippocampal layers (black arrows) and intact intercellular matrix with minimal reactive glial cell infiltrates, on the contrary, the Res group rats revealed severe neuropathic alterations. These modifications included severe neuronal degeneration with abundant records of hyperesenophilic, pyknotic, and structureless pyramidal neurons that lack distinct subcellular details (red arrows) and few dispersed apparent intact cells (black arrows). In addition, severe perineuronal edema accompanied with obviously high reactive glial cell infiltrates (arrowheads) with congested blood vessels (star) were observed. Moreover, the toluidine blue-stained sections of Res group showed decreased count of intact neurons (black arrows) with higher records of degenerated neurons (red arrows), as compared to the control group (F_(3, 24)_ = 472.7, ηp^2^ = 0.9834, p < 0.0001). Interestingly, administration of either Esc or Empa in Res-injected rats demonstrated noticeable neuroprotective efficacy. Improvement of the microscopic picture was visually signified by minimal sporadic records of degenerated neurons in some tissue sections (red arrows), relatively higher figures of intact neurons (black arrows), and milder persistent records of reactive glial cell infiltrates (arrowheads), along with normal appearance of brain matrix.

### Effect of Empa on Res-induced alterations in hippocampal neurotransmitters

**Fig. 5:**
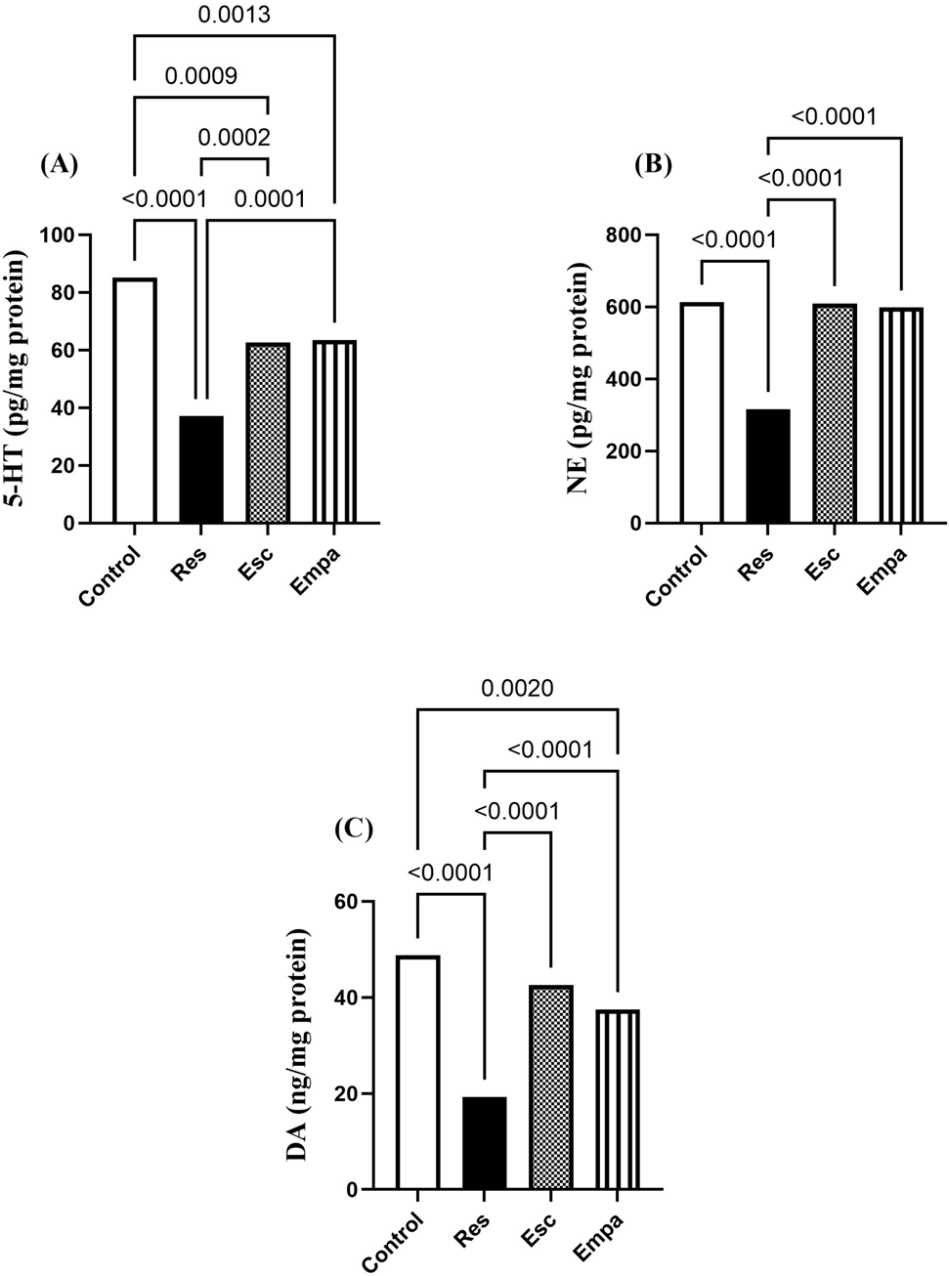
Effects of Empa on Res-induced changes in hippocampal contents of (A) 5-HT, (B) NE, and (C) DA. Data is expressed as mean ± SD (n = 6) using one-way ANOVA followed by Tukey's post-hoc test, (*p* < 0.05). 5-HT; serotonin, DA; dopamine, Empa; empagliflozin, Esc; escitalopram, NE; norepinephrine, Res; reserpine

Dysregulation of neurotransmitters was shown in Res-induced model of depression as evidenced by the significant reductions in the hippocampal contents of 5-HT, NE, and DA, which were almost halved under the effect of Res, when compared to the control group (for 5-HT: F _(3, 20)_ = 32.91, ηp^2^ = 0.8316, p < 0.0001; for NE: F _(3, 20)_ = 125.8, ηp^2^ = 0.9497, p < 0.0001; and for DA: F _(3, 20)_ = 46.01, ηp^2^ = 0.8734, p < 0.0001). Such effects were remarkably reversed by treatment with Empa which produced remarkable upsurges in hippocampal 5-HT, NE, and DA. Of note, Empa produced analogous results to those offered by Esc in terms of its effect on hippocampal neurotransmitters (Fig. 5).

### Impact of Empa on Res-induced alterations in hippocampal *p*-AMPKα1 expression

**Fig. 6:**
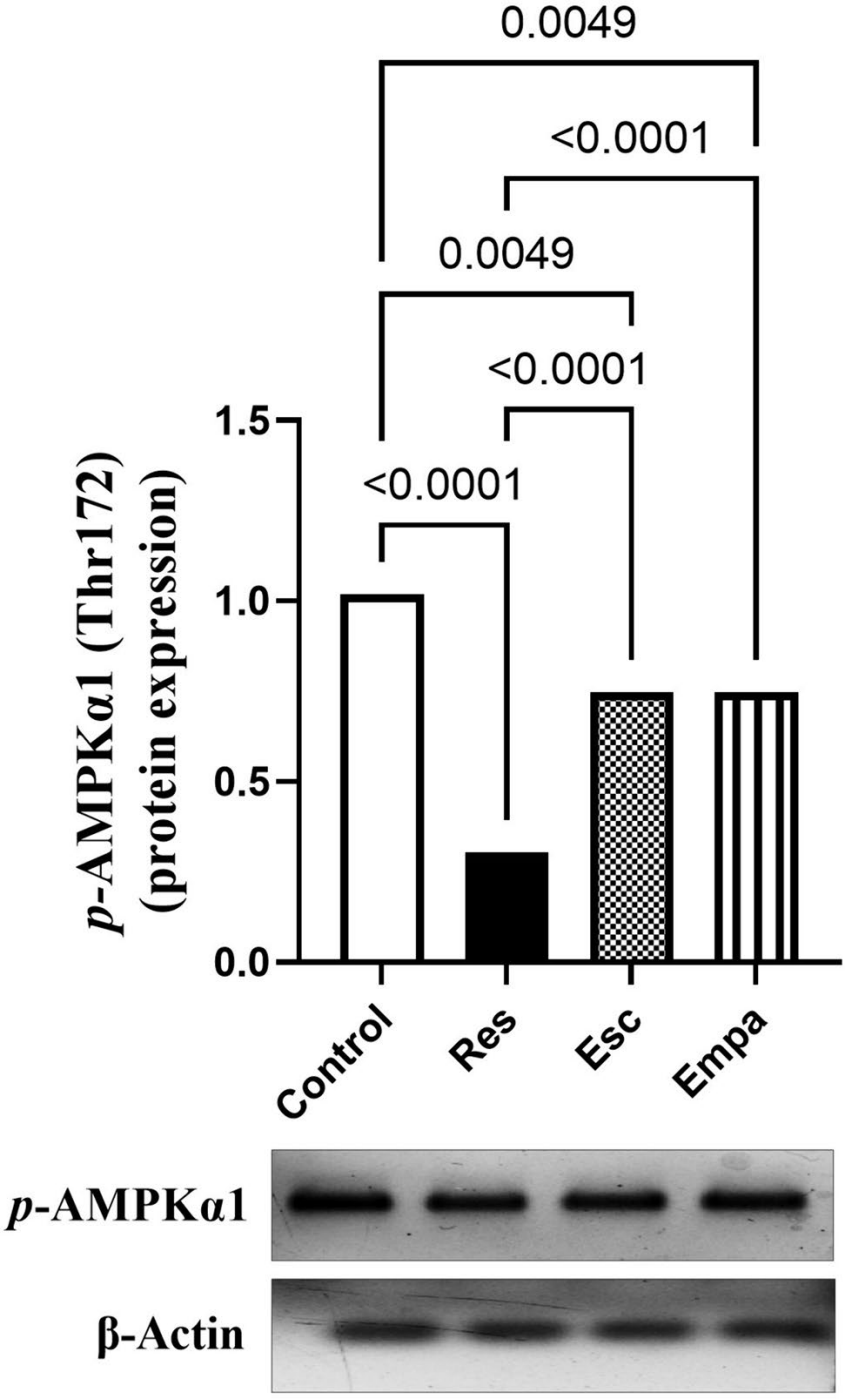
Effects of Res and co-treatments on hippocampal *p*-AMPKα1 (Thr172) gene expression. Data is expressed as mean ± SD (n = 4) using one-way ANOVA followed by Tukey's post-hoc test, (*p* < 0.05). AMPK; adenosine monophosphate-activated protein kinase, Empa; empagliflozin, Esc; escitalopram, Res; reserpine

Hippocampal expression of *p*-AMPKα1 (Thr172) was significantly reduced in rats, as compared to the control group when treated with Res, for it reached one-third the normal values (F _(3, 12)_ = 44.32, ηp^2^ = 0.9172, p < 0.0001). On the contrary, compared to the Res group, rats treated with Esc showed more than two-fold elevation of *p*-AMPKα1. Likewise, *p-*AMPKα1 levels in the hippocampi of Empa-treated rats were significantly higher than those of the Res group but were not significant from Esc (Fig. 6).

### Effect of Empa on Res-induced alterations in hippocampal autophagic regulators

**Fig. 7:**
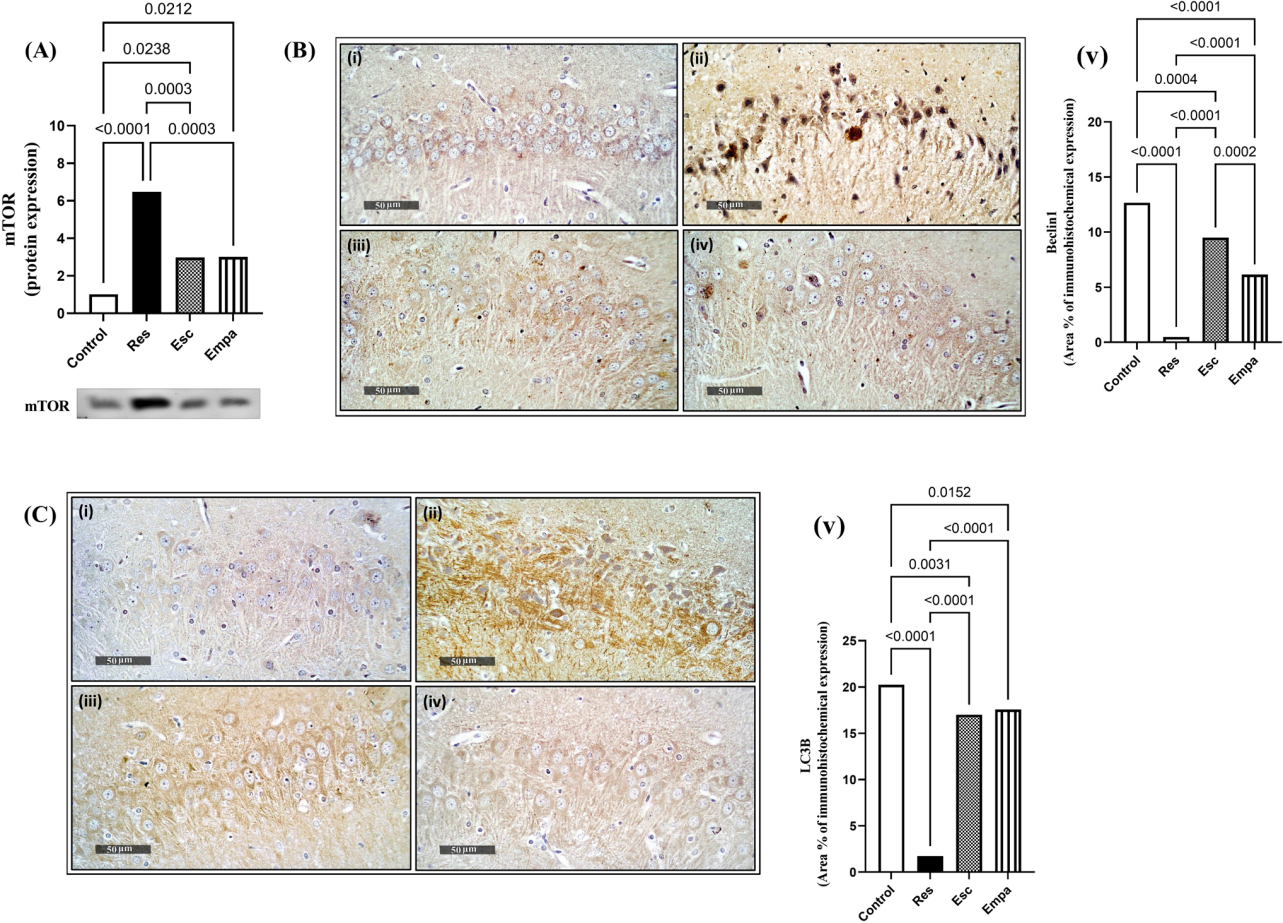
Effects of Empa and Res on hippocampal (A) mTOR expression as well as immunoreactivity of (B) Beclin1 and (C) LC3B, where the representative photomicrographs of Beclin1 and LC3B immuno-staining show microsections of (i) control, (ii) Res, (iii) Res + Esc, and (iv) Res + Empa (n = 6, 400 ×), with (v) showing the percentage area staining for each of Beclin1 and LC3B. For mTOR (n = 4), Beclin1 (n = 6), and LC3B (n = 6), data is expressed as mean ± SD using one-way ANOVA followed by Tukey's post-hoc test, (*p* < 0.05). Empa; empagliflozin, Esc; escitalopram, LC3B; microtubule-associated protein light chain 3, mTOR; mechanistic target of rapamycin, Res; reserpine

Herein, the autophagic markers Beclin1 and LC3B were visualized and quantified by the immunohistochemical technique, while hippocampal expression of mTOR was assessed *via* western blotting analysis. Remarkable dysregulations in hippocampal autophagic machinery were observed through Res, where Beclin1 and LC3B declined and mTOR flourished as compared to the control group (for Beclin1: F_(3, 20)_ = 134.4, ηp^2^ = 0.9528, p < 0.0001; for LC3B: F_(3, 20)_ = 223.7, ηp^2^ = 0.9711, p < 0.0001; and for mTOR: F_(3, 12)_ = 31.01, ηp^2^ = 0.8857, p < 0.0001). These alterations were equally amended by Esc and Empa, except for Beclin1 which was more impacted by Esc than by Empa. In this context, both Esc and Empa significantly replenished the immunohistochemical expressions of Beclin1 and LC3B. Meanwhile, they both reduced the protein expression of mTOR to one-half its value as compared to the Res group (Fig. 7).

### Effect of Empa on Res-induced oxidative burden and neuro-inflammation

**Fig. 8:**
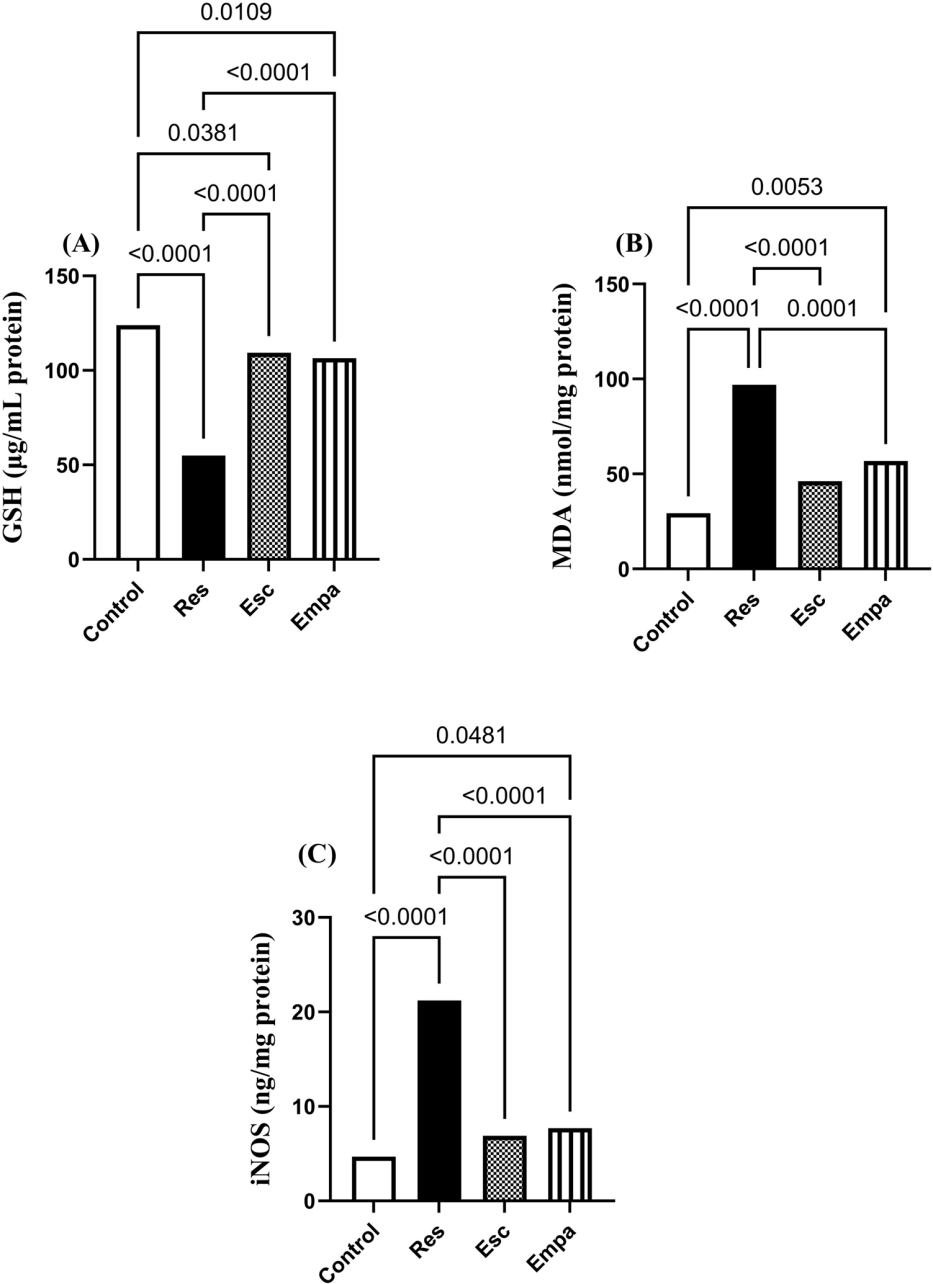
Effects of Empa on Res-induced changes in hippocampal levels of (A) GSH, (B) MDA, and (C) iNOS. Data is expressed as mean ± SD (n = 6) using one-way ANOVA followed by Tukey's post-hoc test, (*p* < 0.05). Empa; empagliflozin, Esc; escitalopram, GSH; glutathione, iNOS; inducible nitric oxide synthase, MDA; malondialdehyde, Res; reserpine

**Fig. 9:**
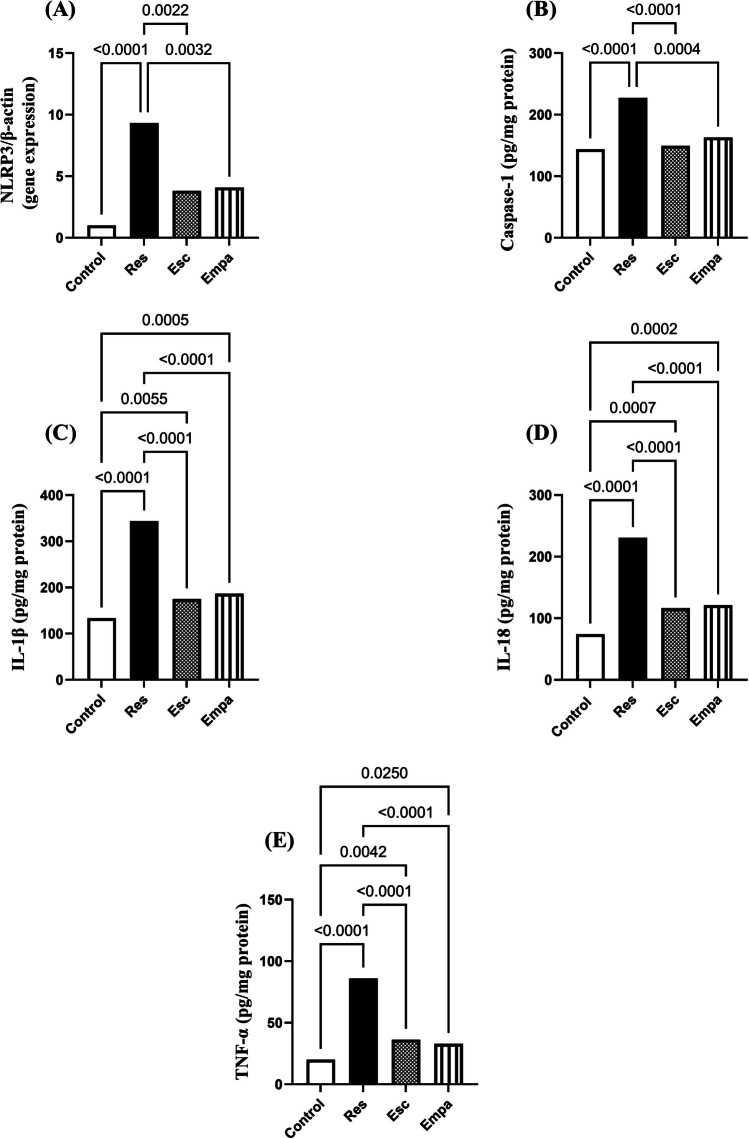
Effects of Empa and Res on hippocampal (A) NLRP3 gene expression in addition to hippocampal contents of (B) caspase-1, (C) IL-1β, (D) IL-18, and (E) TNF-α. For NLRP3 (n = 4), caspase-1, IL-1β, IL-18, and TNF-α (n = 6), data is expressed as mean ± SD using one-way ANOVA followed by Tukey's post-hoc test, (*p* < 0.05). Empa; empagliflozin, Esc; escitalopram, IL; interleukin, NLRP3; nod-like receptor protein 3, Res; reserpine, TNF; Tumor necrosis factor

Administration of Res instigated a profound state of redox imbalance as exemplified by the significant depletion in hippocampal GSH along with marked elevations in MDA and iNOS levels, which came to 45%, 231%, and 354% their normal magnitudes, respectively (for GSH: F_(3, 20)_ = 73.86, ηp^2^ = 0.9172, p < 0.0001; for MDA: F_(3, 20)_ = 31.96, ηp^2^ = 0.8274, p < 0.0001; and for iNOS: F_(3, 20)_ = 98.82, ηp^2^ = 0.9368, p < 0.0001). Empa exerted outstanding antioxidant potentials, equivalent to Esc, which was reflected in the remarkable increment in hippocampal GSH and the mitigation of MDA and iNOS levels, as compared to the Res group (Fig. 8). In line with the aforementioned results, Res promoted a state of inflammatory perturbation driven by the NLRP3 inflammasome pathway. Hence, as compared to the control group, hippocampal gene expression of NLRP3 showed an eight-fold increase in the Res group, with a consequent upsurge in caspase-1 concentration (for NLRP3: F_(3, 12)_ = 18.08, ηp^2^ = 0.8189, p < 0.0001 and for caspase-1: F_(3, 20)_ = 17.85, ηp^2^ = 0.7281, p < 0.0001). The inflammatory disturbance entailed hippocampal contents of IL-1β, IL-18, as well as TNF-α, which reached about 2-, 3-, and 4-folds their normal values, respectively (for IL-1β: F_(3, 20)_ = 143.7 ηp^2^ = 0.9557, p < 0.0001; for IL-18: F_(3, 20)_ = 110.5, ηp^2^ = 0.9431, p < 0.0001; and for TNF-α: F_(3, 20)_ = 97.71, ηp^2^ = 0.9361, p < 0.0001). The NLRP3 inflammasome activation pathway was effectively dampened upon Empa administration resulting in significant decrements in the NLRP3/caspase-1/IL-1β/IL-18/TNF-α cascade versus the Res group. Importantly, both Empa and Esc were equally efficient in terms of their ability to halt the NLRP3 inflammasome overactivation (Fig. 9).

### Effects of Esc and Empa on Res-induced alterations in hippocampal *p*-PKCζ/*p*-NF-κB/BDNF/*p*-CREB pathway

**Fig. 10:**
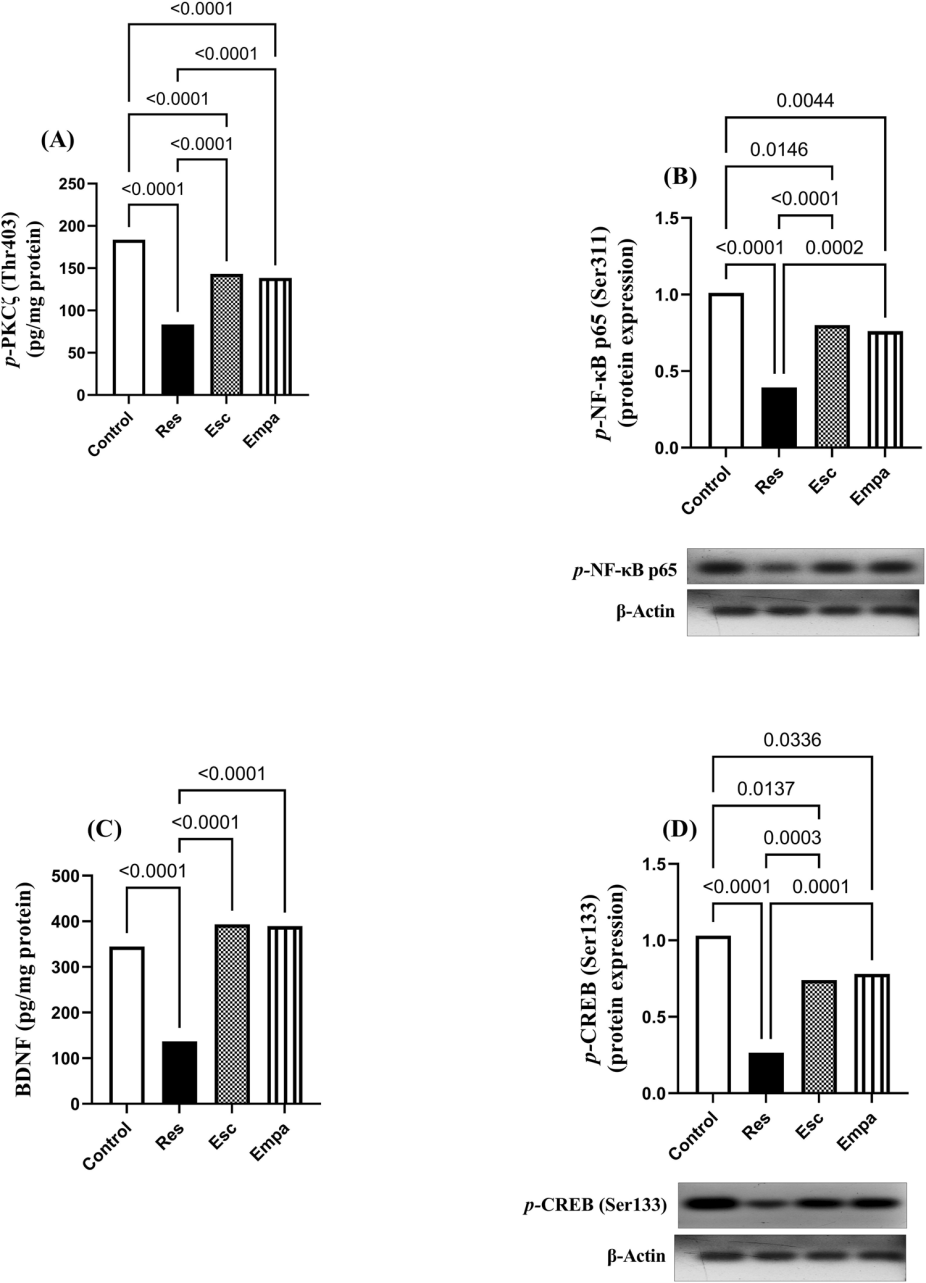
Effects of Empa and Res on hippocampal (A) *p*-PKCζ (Thr403) levels, (B) *p*-NF-kB p65 (S311) expression, (C) BDNF, and (D) *p-*CREB (Ser133) expression. For *p*-PKCζ (n = 6), *p*-NF-kB p65 (n = 4), BDNF (n = 6), and *p*-CREB (n = 4), data is expressed as mean ± SD using one-way ANOVA followed by Tukey's post-hoc test, (*p* < 0.05). BDNF; brain derived neurotrophic factor, CREB; cAMP response element-binding protein, Empa; empagliflozin, Esc; escitalopram, NF-kB; nuclear factor kappa B, PKCζ; protein kinase C zeta, Res; reserpine

Injection with Res induced marked decline in hippocampal *p-*PKCζ (Thr403), *p*-NF-κB p65 (Ser311), BDNF, and *p*-CREB (Ser133), as compared to the control group (for *p-*PKCζ: F_(3, 20)_ = 87.09, ηp^2^ = 0.9289, p < 0.0001; for *p*-NF-κB p65: F_(3, 12)_ = 40.30, ηp^2^ = 0.9097, p < 0.0001; for BDNF: F_(3, 20)_ = 60.65, ηp^2^ = 0.9010, p < 0.0001; and for *p*-CREB: F_(3, 12)_ = 33.38, ηp^2^ = 0.8930, p < 0.0001). Administration of Esc resulted in remarkable increments in the concentrations of *p-*PKC and BDNF, as well as the protein expressions of *p*-NF-κB p65 and *p*-CREB when related to the Res group. Notably, the effects of Empa were comparable to those displayed by Esc, and both drugs outstandingly normalized hippocampal BDNF, as compared to the Res group (Fig. 10).

## Discussion

In light of the growing studies that have been investigating the promising health benefits of SGLT2 inhibitors, the current work puts insights into the neuroprotective potentials of Empa as a driving force for neuroplasticity and resilience. Using Res as a pharmacological insult for depression in rats, Empa was explored as a fine regulator of autophagy and inflammation—two major and opposite processes that largely influence adult neurogenesis. Meanwhile, Esc was implemented as a reference antidepressant drug.

Our results imply that mitigating the depressive behavior in rats by Empa was early recognized in the FST and the TST, in which Empa improved rats' mobility and, convincingly, ameliorated their despair—findings that are constantly reported with standard antidepressants (Chen et al. 2001; Odaira et al. 2019). Also, in a previous study, dapagliflozin, a SGLT2 inhibitor, was able to mitigate the despair behavior of rats exposed to stress in FST (Muhammad et al. 2021). Of note, Res demonstrated a negative outcome on the locomotor function of rats in the OFT, while Empa, on the opposite side, successfully overcame this effect and enhanced the ambulation frequency of rats. Although the influence of depression on locomotor activity has not been consistent among studies due to many variables, Res has been repeatedly linked with motor function deterioration mostly due to DA depletion (Khadrawy et al. 2018; Leao et al. 2015; Lima et al. 2021).

At the biochemical level, the current findings insinuate that hippocampal neuro-inflammation outweighs the autophagic process under Res administration. As previously mentioned, autophagy, the programmed cell survival mechanism, plays a protective role against cell damage in the CNS and throughout the body (Wu et al. 2016; Yazdankhah et al. 2014). In the current work, administration of Empa mitigated Res-induced reduction of hippocampal immunoreactivity of the two autophagy-related proteins, Beclin1 and LC3B which are key agents of early and late autophagic assembly, respectively (Chifenti et al. 2013). These outcomes are in convention with previous reports which demonstrate that rats exposed to chronic mild stress (CMS) acquired depressive symptoms through hindering the autophagic machinery (Yang et al. 2017; Zhao et al. 2017). Likewise, the antidiabetic drug rosiglitazone showed significant antidepressant and neuroprotective properties in unpredictable CMS-triggered depression in mice *via* maintaining autophagy in neurons and mitigating astrocyte-mediated apoptosis (Zhao et al. 2017). Moreover, classical antidepressants such as amitriptyline and citalopram were found to play a crucial role in stimulating autophagy through upregulation of Beclin1 and LC3B (Gulbins et al. 2018; Zschocke et al. 2011). Of note, Beclin1 is fundamental to autophagy-mediated cytoprotection and to opposing apoptotic cell death (Zeng and Zhou 2008). Conversely, our results demonstrate significant decline in mTOR protein expression in response to Empa treatment, which was analogous to the effect generated by Esc. Importantly, inhibition of mTOR pathway has a bona fide role in boosting autophagy (Ravikumar et al. 2004). Additionally, the normal activity of mTOR is pivotal for a sound cognitive function (Uddin et al. 2020). Interestingly, Stanciu and colleagues have established that SGLT2 inhibitors are capable of repressing mTOR, which seems to bring benefits to patients with Alzheimer’s disease (Stanciu et al. 2021). Preclinically, dapagliflozin was found to enhance autophagy through mTOR inhibition in D-galactose-injected/ovariectomized rats (Ibrahim et al. 2022). Moreover, a previous study has revealed that treatment with Empa led to upregulation of AMPK phosphorylation by inhibiting the activation of mTOR (Zheng et al. 2021). In fact, throughout the years, research has been revealing that SGLT2 inhibitors are outstanding AMPK activators (Packer 2020; Yaribeygi et al. 2023; Yu et al. 2022). Of note, phosphorylation of AMPK by upstream kinases at Thr172 can increase its activity by more than 100 folds (Ross et al. 2016).

Besides its chief role in controlling the autophagic reaction, a dynamic interplay exists among AMPK, autophagy, and ROS propagation. In fact, the functional relationship between oxidative stress and autophagy is rather complex. Upon mitochondrial ROS production, redox-sensitive autophagy-related proteins, including AMPK, are rapidly activated to elucidate fast autophagic response. In addition, given that autophagy is a crucial process to eradicate damaged cellular components in response to oxidative stress, it makes sense that ROS are positive effectors for activation of autophagy (Filomeni et al. 2015). Although ROS can initially oxidize, and subsequently, inactivate autophagy-related proteins, ROS ultimately trigger signaling pathways to activate autophagy which in turn suppresses ROS propagation and oxidative damage (Chang et al. 2022). Accordingly, oxidative stress and autophagy regulation can be viewed as a two-way process (Redza-Dutordoir and Averill-Bates 2021). Nonetheless, the story differs with nitric oxide (NO) which can act both as a positive or a negative effector for autophagy. In HeLa cells, NO was found to prevent autophagy by means of halting AMPK and Beclin1 activation, which is in contrast with the well-documented role of NO and nitrative stress in the activation of AMPK in response to DNA damage. This inconsistency is mostly attributed to the fact that NO can range from a pro-survival to a pro-apoptotic molecule depending on its concentration (Filomeni et al. 2015).

Our findings imply that Res administration created a sharp state of oxidative stress in rats' hippocampi, which was revealed by the eminent decline in GSH and the prompt increase in iNOS and MDA. Notably, compared with endothelial and neuronal NOS, iNOS causes nitrative stress and generates more superoxide, which is effective for hindering pathogens but drives inflammation and cell injury as well (Huang et al. 2023). Oxidative stress and neuro-inflammation have been considerably associated with many neurodegenerative diseases, such as Parkinson’s, Alzheimer’s, and Huntington’s diseases (Butterfield 2002; Pizzino et al. 2017; Tripathi et al. 2019). In depression research, the inflammatory cytokines IL-6 and TNF-α have been strongly correlated with the pathology and severity of major depression (Dowlati et al. 2010; Min et al. 2023), and the role of oxidative stress in the progression of depression has long been appreciated (Correia et al. 2023). Noteworthy, the collaborative effects of oxidative/nitrative stress and NLRP3 inflammasome activation result in neuro-inflammation, impaired neurogenesis, and monoamine neurotransmission decline (Correia et al. 2023; Kaufmann et al. 2017). In light of neuro-inflammation, the current results reveal that treatment with Empa plays an important role in dampening the gene expression of the NLRP3 inflammasome, caspase-1 levels, IL-1β/18, and TNF-α. Meanwhile, Empa reversed oxidative stress of the hippocampus, which was observed through the decrements in iNOS and MDA, and alternatively, the potentiation of GSH. In line with our study, Empa was found to prevent cognitive impairment in Alzheimer’s disease as well as in diabetes *via* attenuating cerebral oxidative stress and inflammation (Wicinski et al. 2020; Yaribeygi et al. 2024). In a recent study by Vercalsteren et al. (2024), the authors confirmed that Empa enhanced post-stoke outcomes in diabetic mice partly through hindering neuro-inflammation. In addition, scientists have highlighted the anti-inflammatory and antioxidant potentials of SGLT2 inhibitors, which largely contributes to their health benefits in aging (Schönberger et al. 2023) and in neurological disorders (Tsai et al. 2021). Moreover, researchers have declared that the clinical benefit of antidepressants is partly mediated through alleviating microglial reactivity, oxidative stress, and the release of inflammatory cytokines (Mariani et al. 2022). Importantly, the histopathological and histomorphometric findings reported herein came to support the observed behavioral and biochemical modifications pertinent to either Res or Esc and Empa treatments.

Growing body of evidence have been showing that depressed patients experience reductions in CREB expression and phosphorylation together with BDNF in the hippocampus (Cunha et al. 2006; Zhang et al. 2017). CREB is a transcription factor that has been extensively studied and attested in the pathogenesis of depression and response to therapy. In fact, chronic antidepressant treatment has been verified to upregulate BDNF and its tyrosine kinase receptor B (TrkB) by augmenting phosphorylation of CREB at Ser133 (Tsuchimine et al. 2018). Additionally, it has been reported that BDNF itself stimulates CREB phosphorylation and activation, suggesting that BDNF is capable of regulating its own expression by forming a positive feedback loop (Esvald et al. 2020; Finkbeiner et al. 1997). Noteworthy, one of the involved enzymes in activating CREB is PKC. In its active form, CREB plays a fundamental role in CNS development, cognitive function, and neural survival (Ren et al. 2011). In the present study, Empa administration restored hippocampal *p-*CREB (Ser133) expression and BDNF concentration in Res model of depression, and thus, affording comparable effects to those of Esc. Empa has been previously reported to replenish BDNF in diabetic obese mice and to improve their cognitive function (Lin et al. 2014; Piątkowska-Chmiel et al. 2023). In addition, SGLT2 inhibitors have been found to relieve hyperuricemia in diabetic mice (Lu et al. 2020), and obesity in high fat-fed mice *via* enhancing CREB phosphorylation (Yang et al. 2021). Indeed, the aforementioned effects are not independent from AMPK. Through activating the atypical PKCζ signaling, AMPK plays an indispensable role in boosting hippocampal neurogenesis (Wang et al. 2012). Intriguingly, AMPK has been reported to mediate exercise-induced BDNF augmentation in hippocampi of stressed mice (Kim and Leem 2016). In the same context, PKCζ-mediated BDNF potentiation is thought to arise under the influence of NF-κB (Odaira et al. 2019). In fact, the role of NF-κB in BDNF secretion has not been thoroughly investigated, although it represents an attractive point for research. To be more specific, phosphorylation of NF-κB p65 at Ser311 residue, rather than the famous Ser536 site, has been shown to play a fundamental role in neuronal survival, defending neurons against oxidative stress, and extenuating pyramidal neuronal death (Kim et al. 2013). Importantly, phosphorylation of NF-κB p65 at Ser311 specifically occurs upon PKCζ activation and phosphorylation at Thr403 (Wang et al. 2012). Nonetheless, some authors have documented conflicting results. In this context, Yi et al. (2021) argue against sustained AMPK activation which has negatively affected BDNF/TrkB signaling due to mTOR inhibition in stressed mice. Additionally, NF-κB has been well reported to upregulate NLRP3 expression, which might contradict the current results (Song et al. 2017). Although NF-κB p65 with or without Ser536 phosphorylation has been confirmed to induce NLRP3 expression and to provoke the inflammatory response (Boaru et al. 2015; Perkins and Gilmore 2006) the correspondence between *p*-NF-κB p65 Ser311 and NLRP3 has not been addressed so far.

## Conclusions

The current study emphasizes the role of Empa in neuroprotection and neural resilience potentiation in the Res animal model of depression. Mastered by AMPK activation, the SGLT2 inhibitor is thought to fine regulate the triad of autophagy, inflammation, and oxidative stress in the brain. Deeper evaluation revealed the ability of Empa to rebalance the disturbed autophagy/inflammation dynamics through manipulation of the autophagic machinery components: mTOR/Beclin1/LC3B and attenuation of the NLRP3 inflammasome cascade. Consequently, reinforcement of hippocampal neurogenesis was demonstrated as a valuable outcome of Empa treatment. This latter effect is thought to be mediated partly through AMPK which orchestrates PKCζ phosphorylation and highlights an unusual role for NF-κB in boosting BDNF production—an outcome that necessitates NF-κB phosphorylation at Ser311 upon interaction with PKCζ.

## Supplementary Information

Below is the link to the electronic supplementary material.

Supplementary file1 (PDF 512 KB)

## Data Availability

The original contributions presented in the study are included in the article. Further inquiries can be directed to the corresponding author.
