# Supplementary material for: Empagliflozin-activated AMPK elicits neuroprotective properties in reserpine-induced depression via regulating dynamics of hippocampal autophagy/inflammation and PKCζ-mediated neurogenesis
Source: Psychopharmacology (Berl). 2024 Aug 19;241(12):2565–84. doi: 10.1007/s00213-024-06663-0 (PMC11569022; doi:10.1007/s00213-024-06663-0)
Supplement: Supplementary file 1 — Supplementary file1 (PDF 512 KB) [file 213_2024_6663_MOESM1_ESM.pdf]

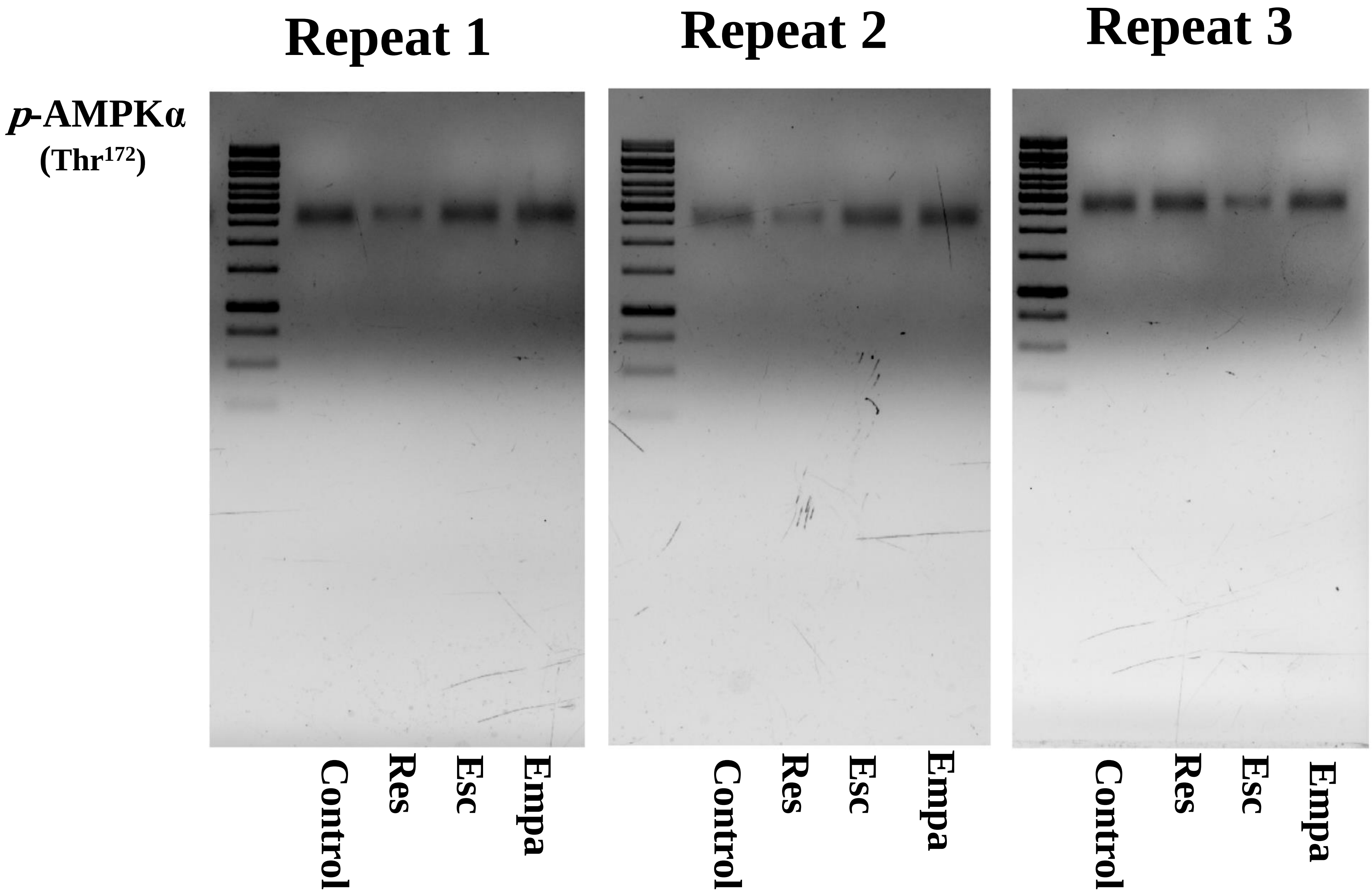
Repeat 1
Repeat 2
Repeat 3
p-AMPKα
(Thr172)
Control
Res
Esc
Empa
Control
Res
Esc
Empa
Control
Res
Esc
Empa

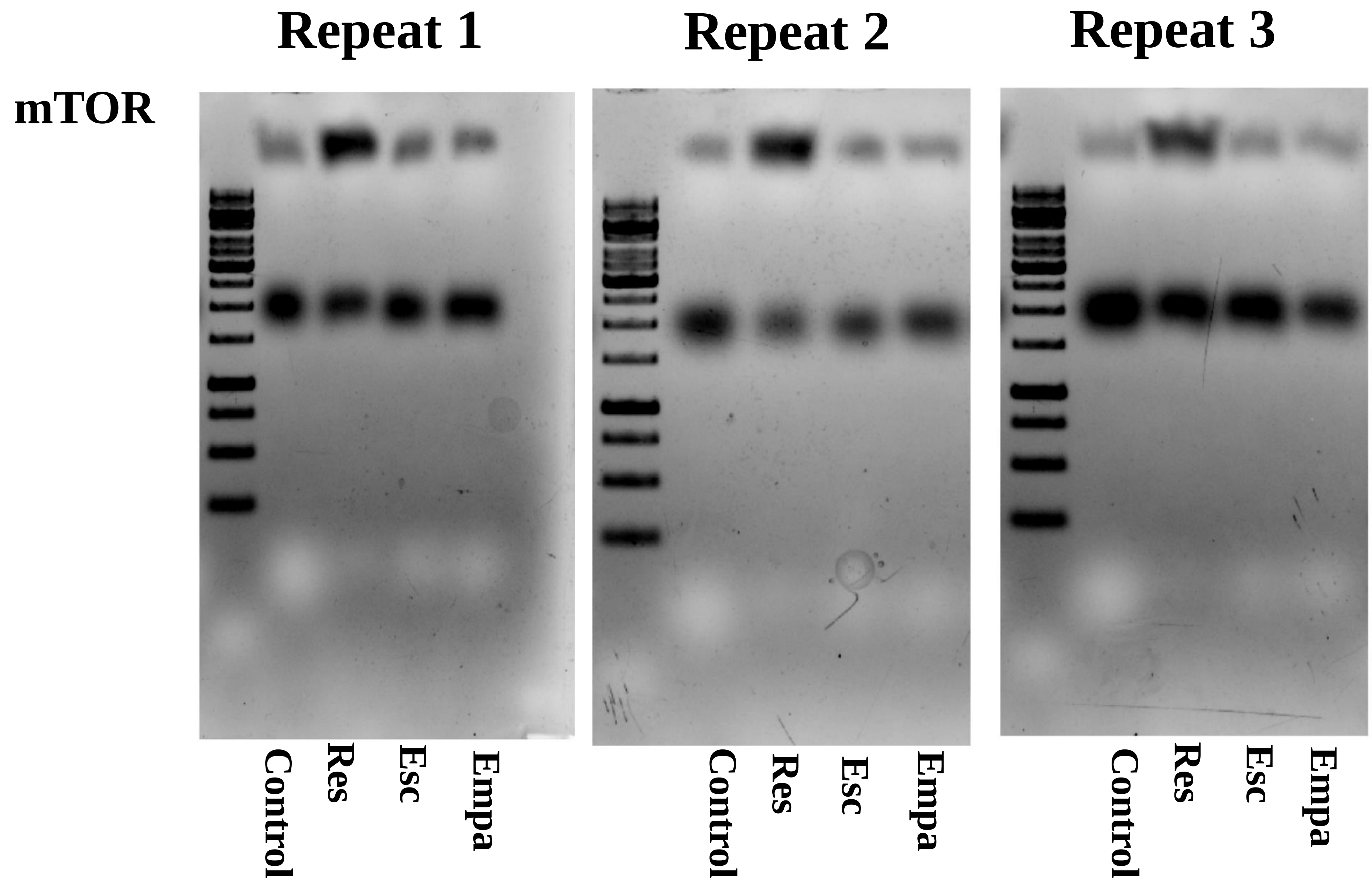
Repeat 1
Repeat 2
Repeat 3
mTOR
Control
Res
Esc
Empa
Control
Res
Esc
Empa
Control
Res
Esc
Empa

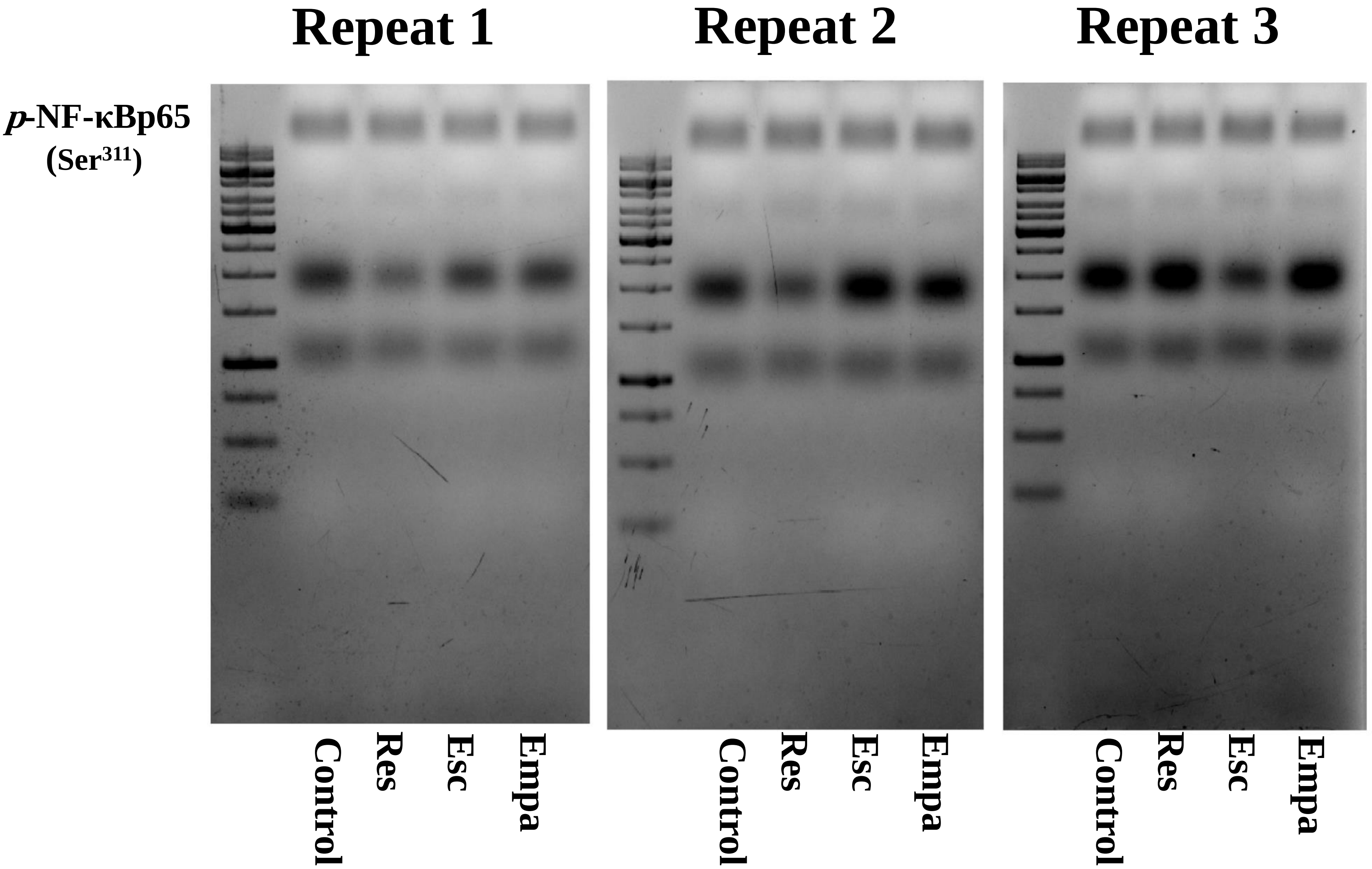
Repeat 1
Repeat 2
Repeat 3
p-NF-κBp65
(Ser311)
Control
Res
Esc
Empa
Control
Res
Esc
Empa
Control
Res
Esc
Empa

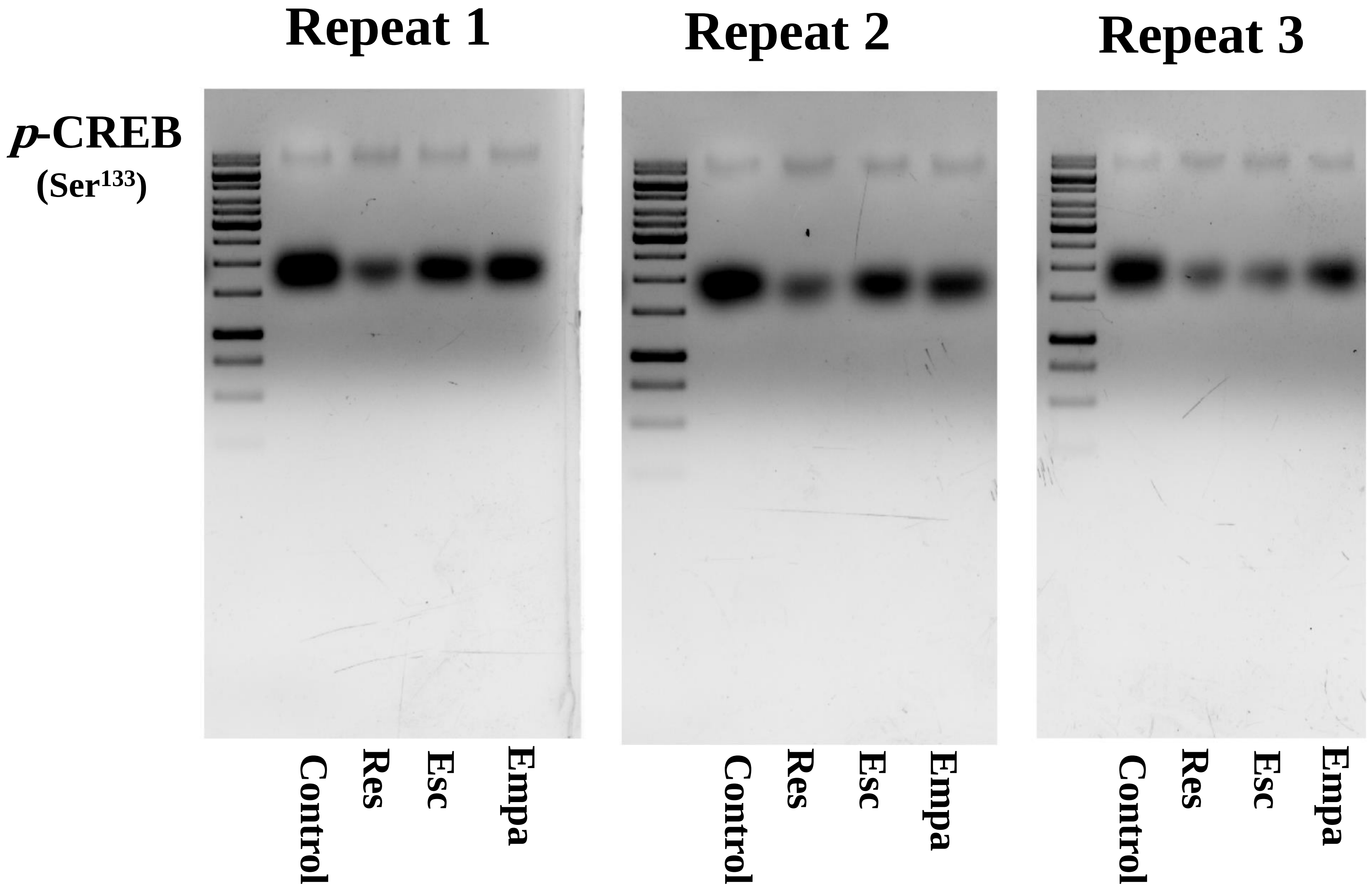
Repeat 1
Repeat 2
Repeat 3
p-CREB
(Ser133)
Control
Res
Esc
Empa
Control
Res
Esc
Empa
Control
Res
Esc
Empa

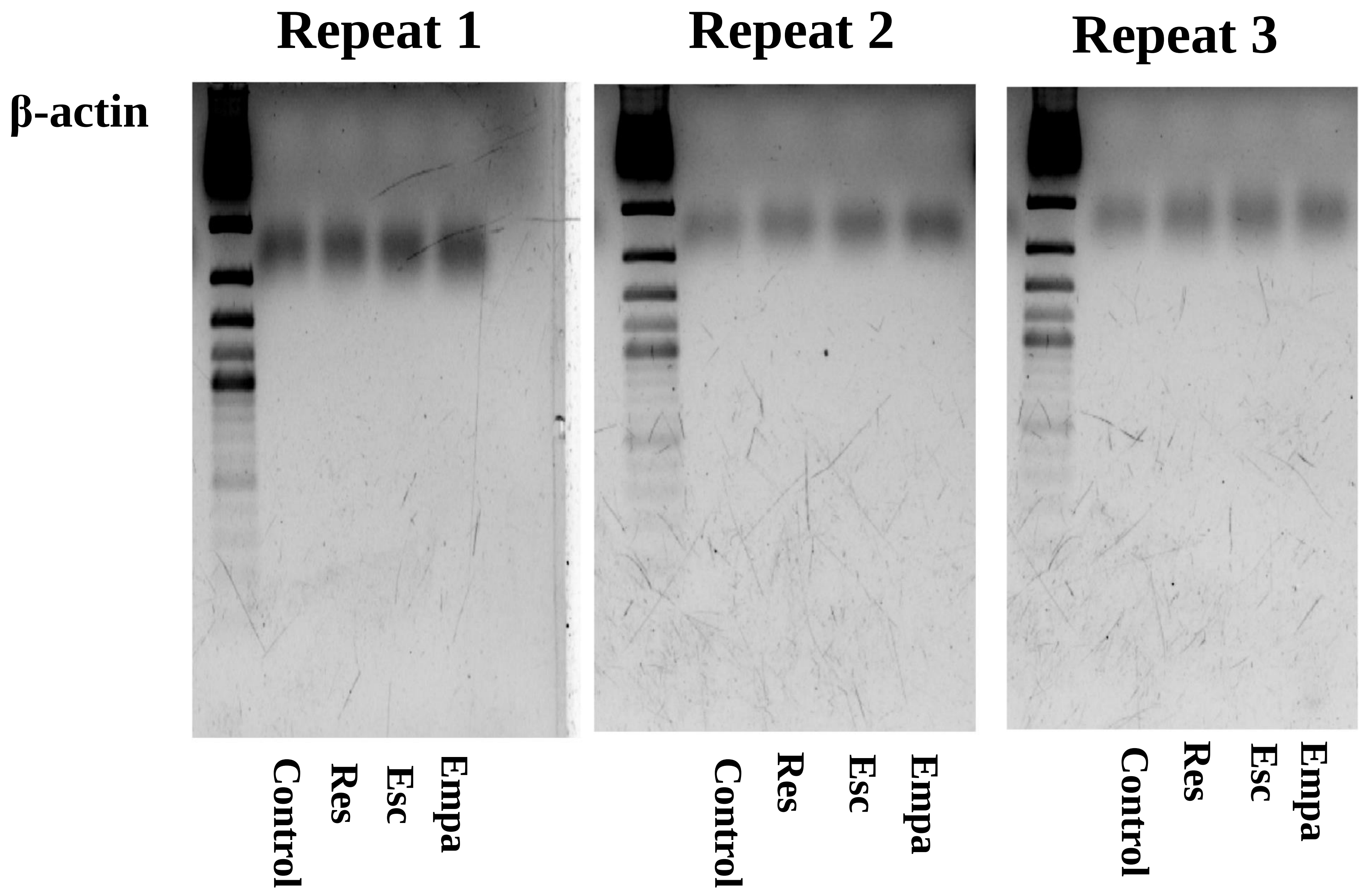
Repeat 1
Repeat 2
Repeat 3
β-actin
Control
Res
Esc
Empa
Control
Res
Esc
Empa
Control
Res
Esc
Empa
